# The role of nursing communication: A critical interpretive synthesis

**DOI:** 10.1016/j.ijnsa.2025.100373

**Published:** 2025-06-27

**Authors:** Julie B. Grant, Jacqueline Jones, Carey Candrian, Kathleen S. Oman, Sean M. Reed

**Affiliations:** a College of Nursing, University of Colorado Anschutz Medical Campus, Aurora, 80045, USA; b Division of General Internal Medicine, University of Colorado Anschutz Medical Campus, Aurora, 80045, USA

**Keywords:** Communication, Patient care, Nursing communication, Critical interpretive synthesis, Conceptual framework, Nursing presence

## Abstract

**Aim:**

The primary aim of this paper is to explore and interpret the *role of nursing communication in patient care*. The secondary aim is to identify conceptual inconsistencies in the existing literature and synthesize these insights with our previous interpretations to develop a conceptual framework that clarifies the role of nursing communication, thereby providing a nursing-specific foundation for developing communication skills.

**Background:**

The need for *effective nursing communication* is widely emphasized within the discipline of nursing, and despite the widespread use of that phrase, its role in patient care and the nursing profession remains nebulous. Nursing communication's meaning and value have become more equated with financial indicators than the nurse-patient relationships central to the phenomenon.

**Methods:**

A Critical Interpretive Synthesis approach was used to interpret the role of nursing communication in patient care. Electronic searches of *PubMed, EBSCOhost, Cochrane Library, PsycINFO, Communication & Mass Media Complete, Web of Science, Embase,* and *Google Scholar* were conducted between January 2024 and March 2024. A CIS seven-phase process guided the literature synthesis and formation of a conceptual framework.

**Results:**

Five synthetic constructs clarify the role of nursing communication and contribute to the final conceptual framework: 1) ongoing holistic engagement, 2) humble guide supporting the patient's itinerary, 3) effective information exchange, 4) maintaining nursing individuality within an existing system, and 5) nursing presence.

**Conclusion:**

The role of nursing communication emphasizes maintaining a nursing presence that communicates high-quality physical care and ongoing holistic engagement centered around the patient's values, needs, and wishes. This conceptual framework prioritizes the unique nurse-patient communicatory relationship and gives nurse researchers, policymakers, and educators a new way of thinking about and prioritizing the role of nursing communication.

**Contribution of the Paper**

**What is already known**

• Critical Interpretive Syntheses build theory by dialectically examining existing research
• Nursing communication is learned and innate, combining trainable skills and nurse individuality

**What this paper adds**

• Nursing communication prioritizes patient values to holistically address their needs
• A caring nursing presence communicates support and empathy that transcends cultures and systems
• The role of nursing communication is unique and requires its own theory and research

Alt-text: Unlabelled box

## Rationale and aims of the study

The term *nursing communication* and the need for *effective nursing communication* is widely emphasized within the discipline of nursing and related health fields (Kerr et al., 2020). Despite the widespread use of the term *nursing communication*, its role in patient care and the nursing profession remains nebulous, with its meaning and value equated more with financial indicators such as Hospital Consumer Assessment of Healthcare Providers and Systems (HCAHP) scores than with the nurse-patient relationships central to the phenomenon (Höglander et al., 2023; Trotta et al., 2020; Watts et al., 2021). Therefore, the primary aim of this Critical Interpretive Synthesis is to explore and interpret the *role of nursing communication in patient care*. Our secondary aim is to identify conceptual inconsistencies in the existing literature and synthesize these insights with our previous interpretations to develop a conceptual framework that clarifies the role of nursing communication, thereby providing a nursing-specific foundation for developing communication skills. For the purposes of this research, the role players in nursing communication include nurses, patients, and any other individuals with whom the nurse communicates, verbally or nonverbally, in ways that influence patient care.

## Background

Nursing communication is an essential building block for patient care, trust, and comfort. Effective communication in nursing is touted as one of the most critical aspects of nursing care, with many publications highlighting its potential to improve patient safety and satisfaction scores (Gutiérrez-Puertas et al., 2020; Kerr et al., 2020). The past decades have shown a surge of research seeking to improve communication in nursing through policy, practice, and education (Gutiérrez-Puertas et al., 2020; Kerr et al., 2020). Patient-reported complaints frequently cite ineffective or failed communication as a central concern (Elliott et al., 2009; Reader et al., 2014), and an estimated 70% to 80% of healthcare errors and sentinel events are attributed to poor communication among healthcare teams (The Joint Commission, 2023). Additionally, the growing spread of health-related misinformation has further complicated effective communication in healthcare settings (Andrews, 2019; Wang et al., 2019; Waszak et al., 2018). However, the concept of nursing communication is often oversimplified in both literature and practice and is frequently evaluated through narrow financial metrics, such as HCAHPS scores, rather than grounded in the foundational nurse-patient relationships it is intended to reflect (Höglander et al., 2023; Trotta et al., 2020; Watts et al., 2021). For instance, the HCAHPS question, “Before giving you any new medicine, how often did hospital staff describe possible side effects in a way you could understand?” (Hospital Consumer Assessment of Healthcare Providers and Systems, 2025, p. 3) is commonly used as a proxy for effective nursing communication. Yet, this measure fails to capture the complexity and nuance of everyday nurse-patient interactions—such as a nurse gently redirecting a confused patient in the hallway, responding to calls while already engaged with another patient, or simply sitting at the bedside listening to a patient’s story as a way to ease their emotional or physical distress (Trotta et al., 2020).

While hundreds of research studies hypothesize what can be done to enhance effective communication in nursing, almost none provide basic conceptualizations of nursing communication from which to base their research or analysis (Afriyie, 2020; Gutiérrez-Puertas et al., 2020; Höglander et al., 2023; Kerr et al., 2020). The term communication by itself has a variety of definitions. The dictionary defines communication as "a process by which information is exchanged between individuals through a common system of symbols, signs, or behaviors" (Merriam-Webster, 2023). The American Association of Colleges of Nursing expands on the dictionary definition by including “intentionality, mutuality, partnerships, trust, and presence" (American Association of Colleges of Nursing, 2023, p. para. 1). This variety of definitions highlights the need for clarity on the specific role of nursing communication in patient care.

## Methods

### Study design

A Critical Interpretive Synthesis approach was used to analyze the role of nursing communication because of our broad question of interest that explores and interprets the role of nursing communication in patient care, the scope of potentially applicable literature, and the heterogeneous nature of the existing literature that informs this work (Dixon-Woods et al., 2006). The complexities surrounding nursing communication require an analysis beyond empirical effectiveness research or concept analyses alone and necessitates the synthesis of multiple forms and levels of evidence. While traditional systematic reviews typically address questions of effectiveness, a Critical Interpretive Synthesis aims to support the generating of frameworks and middle-range theories with explanatory power, thereby enhancing the reader’s understanding and interpretation of complex concepts and phenomena (Dixon-Woods et al., 2006; Flemming, 2010; Schick-Makaroff et al., 2016). As we are explicitly attempting to avoid analyzing the "effectiveness" of nursing communication and instead are interpreting the role of nursing communication, the goals of this research and Critical Interpretive Synthesisalign. Furthermore, maintaining a focus on creating a framework throughout the Critical Interpretive Synthesisprocess reminded us to continually consider how the emerging concepts interacted, aligned, and diverged, thereby informing the structure and coherence of the final synthesis.

### Compass question

The initial phase of a Critical Interpretive Synthesis involves the development of a broad, exploratory question, referred to as the compass question, that serves to orient and guide the analytical process. This question is intentionally open-ended to facilitate the inclusion of diverse sources, perspectives, and forms of evidence. In this study, the compass question was derived from the overarching aim of exploring and interpreting the role of nursing communication in patient care, while also addressing significant conceptual inconsistencies in the literature. The research specifically sought to understand how nursing communication is conceptualized within the context of patient care, with the ultimate objective of contributing to the development of a conceptual framework to inform future nursing practice, research, and theoretical advancement. Accordingly, the compass question guiding this synthesis was: How is the role of nursing communication in patient care conceptualized?

### Literature search strategy

A Critical Interpretive Synthesismandates a broad search strategy (Depraetere et al., 2021; Flemming, 2010). Electronic database searches of *PubMed, EBSCOhost, Cochrane Library, PsycINFO, Communication & Mass Media Complete, Web of Science, Embase,* and *Google Scholar* were conducted between January 2024 and March 2024. Search results were limited to the last 10 years. Employed keywords were selected based on pertinence to the research topic rather than mesh terms, as the latter yielded excessive, irrelevant results lacking the necessary specificity. Database searches utilized the Boolean phrase ((("nurse communication" [Title/Abstract]) OR ("nursing communication" [Title/Abstract])) OR ("rn communication" [Title/Abstract])) OR ("communication in nursing" [Title/Abstract]). The Google Scholar searched the phrase “nursing communication” in the article title. Further data was compiled from website searches and reference chaining. The choice to include non-peer-reviewed grey literature stems from Critical Interpretive Synthesis' emphasis on casting the widest net possible to understand how individuals perceive the role of nursing communication outside research and academic settings.

**Fig. 1:**
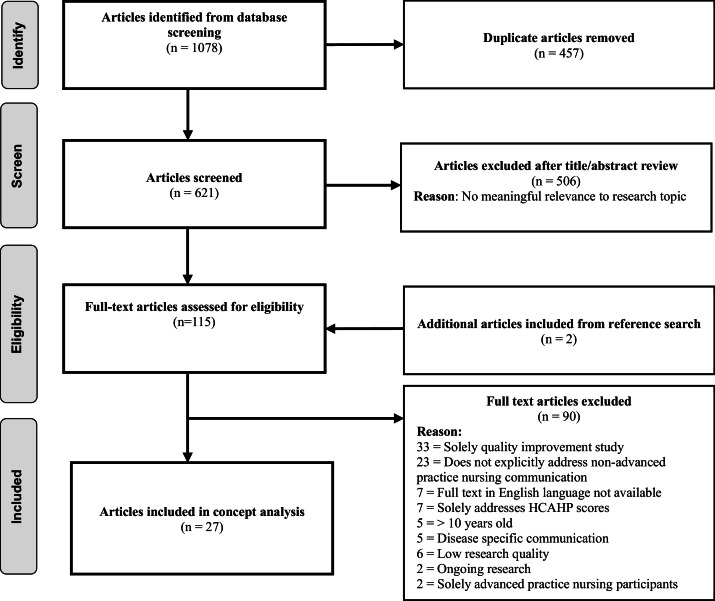
PRISMA diagram of literature search.

All quality appraisal and data abstractions were transposed to Covidence, which served as an organizational tool for the literature synthesis, quality appraisals, and Preferred Reporting Items for Systematic reviews and Meta-Analyses (PRISMA) review (Page et al., 2021) (Fig. 1). The data abstractions documented in Covidence were uploaded into Atlas.ti as primary documents. Atlas.ti assisted with the organization of codes, subcodes, themes, and memos and helped visualize patterns and relationships. However, Atlas.ti was used solely to augment the primary author's interpretive work, and no artificial intelligence functions were used.

### Quality appraisal

Quality appraisals were conducted for all studies, and any articles deemed so low quality as not to provide meaningful contributions were excluded. The Effective Public Health Practice Project Quality Assessment Tool for Quantitative Studies assessed quantitative research (Effective Public Health Practice Project, 2010, p. E50). Qualitative research was appraised using McMaster University's Guidelines for Critical Review Form: qualitative studies (version 2.0) (Letts et al., 2007). Quantitative and empirical syntheses were assessed using the Assessment of Multiple Systematic Reviews (AMSTAR) (Shea et al., 2007). Two outside reviewers trained in quality appraisal methods randomly selected 10 percent of the research articles. These reviewers conducted blinded quality appraisals that were compared to the primary reviewer’s to ensure consistency and methodological reproducibility (Flemming, 2010). Outside quality appraisers did not participate in synthesizing, interpreting, or re-contextualizing the data.

### Inclusion and exclusion

Critical Interpretive Synthesis necessitates flexible inclusion selection criteria (Depraetere et al., 2021; Dixon-Woods et al., 2006). Inclusion criteria for database searches were articles published in the last 10 years, explicitly addressing “nursing communication” or “communication in nursing” in the title or abstract, and full text available in English. Articles were excluded if they were quality improvement interventions, did not address the role of registered nurses, were solely associated with HCAHP scores, the communication addressed in the research was overly disease-specific, the study was ongoing, qualitative syntheses, and research with deficient quality.

### Data analysis and synthesis

**Fig. 2:**
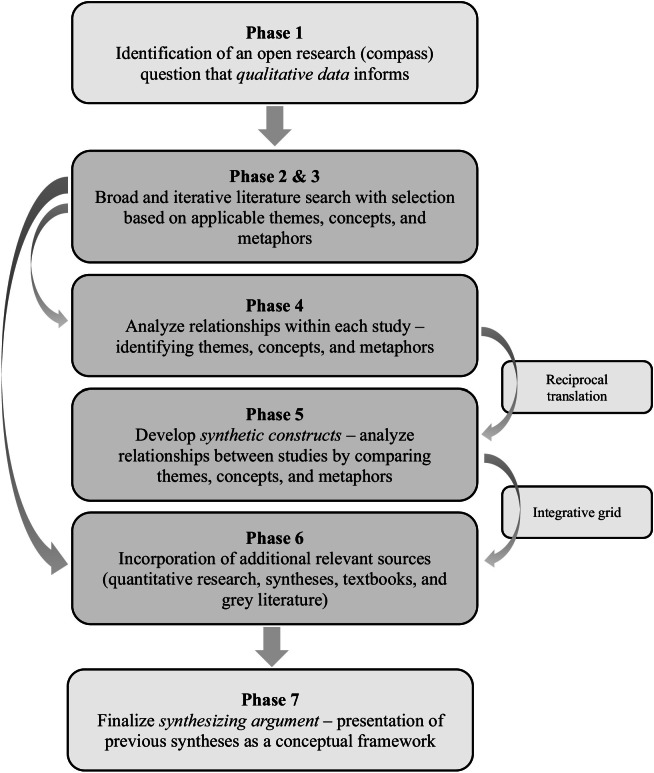
Seven-phase process of a critical interpretive synthesis.

The synthesis portion of the Critical Interpretive Synthesis used an adapted meta-ethnography method for identifying themes and concepts that subsequently guide a uniquely Critical Interpretive Synthesis seven-phase process for generating final outputs (Fig. 2) (Depraetere et al., 2021; Flemming, 2010; Noblit and Hare, 1999).

Phase one identified an open research question that qualitative data could inform. Phase two was a broad literature search. Phase three included a literature selection process that based its article selections on applicable themes, concepts, and metaphors. In phase three, articles were first read to understand the paper in relation to itself and its context before comparing it to other sources (Flemming, 2010). Phase four expanded on phase three to compile definitions, concepts, themes, and metaphors from individual papers. Phase five built upon the information complied in phase four and translated the studies into one another by comparing themes, concepts, and metaphors to produce a concentrated, not diminished, account of the studies' content (Flemming, 2010). Phases four and five utilized a modified reciprocal translation technique that explicitly incorporated metaphors, primary themes, and subthemes (Table 1) (Melendez-Torres et al., 2015).

**Table 1:** Reciprocal translation.

Number	Codes	Sub-Codes	Primary Study Themes or Subtheme (ST) Exemplar Quote (EQ) Author Quote (AQ) Researcher Memo (RM)	Document	NOTES / MEMO:
11:41	Effectively exchange knowledge and information	Translator	As a patient advocate, they communicate with their medical colleagues the needs and wishes of the patient/family" (p. 917).	Kelly 2023	
14:31	Holistically engage with the patient and their loved ones	Comfort, reassurance, and trust	By being "real" with the patient or caregiver rather than treating him or her as fragile, the nurse communicated respect (p. 582) (AQ)	Oliver 2019	
16:25	Holistically engage with the patient and their loved ones	Comfort, reassurance, and trust	nurses voices should be relied upon to promote communication in patient care that focuses on safety and optimizing clinical outcomes while decreasing uncertainty and distress. (AQ)	Simonovich 2021	
6:28	Holistically engage with the patient and their loved ones	Comfort, reassurance, and trust	Emotionally laden patient and caregiver concern statements require the hospice nurse to compose an answer that is empathetic, accurate, and meets patient and caregiver needs. In other words, nurses must use a patient-centered approach when providing end-of-life communication. (AQ)	Clayton 2014	Techniques?
5:51	Holistically engage with the patient and their loved ones	Comfort, reassurance, and trust	Very few of the study participants discussed their doubts, fears and uncertainties with the nurses. Overall, there was a tendency for the participants to discuss their fears with their doctors and families rather than the nurses. (AQ)	Alshammari 2022	Contradictory quote
3:48	Holistically engage with the patient and their loved ones	Comfort, reassurance, and trust	if participants were frustrated because they had to be woken from sleep, they appreciated the nurses' apologies and clear rationale for waking them. The apologies lessened frustration, allayed dissatisfaction, and demonstrated courtesy and respect. (AQ)	Trotta 2020	

*Note.*Table 1 provides an abbreviated example of the reciprocal translation utilized in this study. It is excerpted from the comprehensive reciprocal translation table developed for the data analysis.

Using concepts and themes derived from the previous reciprocal translation, phase six incorporated additional relevant sources such as quantitative syntheses, pertinent quantitative research, textbooks, and grey literature using an integrative grid (Table 2) (Depraetere et al., 2021; Flemming, 2010). The primary and secondary themes identified in the reciprocal translation were at the top of the grid. Each grid row represented a high-quality quantitative synthesis plus applicable stand-alone quantitative research. The intersecting cells were populated by the quantitative research findings that either supported or refuted the initial themes and subthemes. This integrative grid allowed for testing initial themes and subthemes and showed how they supported or contrasted with previous quantitative research findings, essentially testing the emerging framework using existing quantitative research. We finalized the initial themes and subthemes into synthetic constructs with this new information. Synthetic constructs result from transforming the underlying evidence into a new conceptual form and creating a synthesizing argument (Flemming, 2010). Phase seven presented the previous syntheses, synthetic constructs, and synthesizing argument as a conceptual framework (Flemming, 2010).

**Table 2:** Integrative grid.

Empirical Syntheses and Quantitative Studies		Synthetic Constructs Resulting from Reciprocal Translation							
		Ongoing Holistic Engagement			Humble Guide Supporting the Patient's Itinerary		Effective Information Exchange		Msc.
Author/Year	Title	Engaging in Conversation	Anticipation of Needs	Reframing Hope	Foster Cultural Safety	Mediate Toward Patient's Wishes	Factual and Logistical Information Exchange	Translator	
Höglander et al. 2023	Registered nurse-patient communication research: an integrative review for future directions in nursing research	Patients talked more when RNs used open ended questions and communication loops	Patients often vague or unclear in expressions of their concerns, making communication skills of RN vital	Patients more active in conversation when RN's expressed positive emotions, understanding, or agreement and used small talk	Including family and loved ones promoted trust in the RN-patient relationship	-	When RN was in an instructor role, RN talking dominated conversation	Communication physical care - Psychosocial, positive emotions, partner	-
	-	RN non-verbal communication aided in communicating care, concern, warmth, empathy, and demonstrated friendship	-	-	*RNs distanced themselves through one-way communication*	*Agenda for communication primarily set by RN*	Communication seldom explored patients existential and psychosocial concerns	*RNs distanced themselves through medical jargon*	-
Gutiérrez-Puertas et al., 2020	Educational interventions for nursing students to develop communication skills with patients: a systematic review	Simulation was the most common intervention to measure nurse-patient communication	-		-	-	No valid and reliable measure for effective patient centered communication	-	Multiple definitions for patient-centered communication -Therapeutic communication, nurse-patient communication, or interpersonal communication
Kerr et al., 2020	The effectiveness training interventions on nurses' communication skills: a systematic review	-		-	Communication research needs to address structural and cultural barriers in health services.	-	Direct impact of edu interventions on RN communication difficult to measure b/c of non-standardized outcome measurement tools	-	Majority of research focused on cancer patients. More research needed in acute, chronic illness, aged care and community settings

*Note.*Table 2 provides an abbreviated example of the integrative grid utilized in this study. It is excerpted from the comprehensive integrative grid developed for the data analysis.

## Results

### Search results and article selection

The electronic database search yielded 1078 articles. After removing duplicate articles and reviewing the initial title and abstract, 115 articles remained eligible for full-text review. During the full-text review, 90 publications lacked inclusion criteria, while two additional articles were identified for inclusion from reference chaining. Subsequently, 27 articles remained applicable for inclusion in this Critical Interpretive Synthesis (Table 3). Included articles consisted of *17 qualitative studies* (Adams et al., 2014; Alshammari et al., 2022; Clayton et al., 2014; Crawford et al., 2017; Forbes and Evans, 2022; Forbes et al., 2020; Holm et al., 2021; Hudson et al., 2019; Kelly et al., 2023; Li et al., 2020; Montgomery et al., 2017; Oliver et al., 2019; Paulsen et al., 2023; Pecanac and King, 2019; Simonovich et al., 2021; Trotta et al., 2020; Wittenberg et al., 2016), *seven quantitative studies* (de Rezende et al., 2015; Kim and Sim, 2020; Myers et al., 2020; Reblin et al., 2019; Saraswasta et al., 2021; Wittenberg et al., 2016; Wune et al., 2020), and *three quantitative or empirical syntheses* (Gutiérrez-Puertas et al., 2020; Höglander et al., 2023; Kerr et al., 2020). Only one author who published two of the included research articles provided a guiding nursing theory or framework (Wittenberg et al., 2018; Wittenberg et al., 2017). While many studies highlighted their chosen communication techniques, few justified how those techniques could be incorporated with an existing nursing role, other than to say the nurse could be further educated on the method (Kerr et al., 2020).

**Table 3:** Role of nursing communication literature matrix.

Author/ Year	Title	Country	Purpose	Study Design	Theory/ Framework	Method(s)	Participants	Summary of Findings
**Crawford 2017**	Culture shapes nursing practice: Findings from a New Zealand study	New Zealand	Investigate nurses' and parents' experiences of communication about parental emotions in a hospital setting – focus on the environmental and cultural context	Qualitative - Ethnography	None	Participant observation Formal interviews Informal interviews	n=10 nurses n=10 patients	- -Parents' experiences of being in the unit – effort to understand what was going on in the unit for themselves and their children. - Parents' had roller coaster of emotions Parents' expectations of nurses were that they would engage and connect. - The parent was reluctant to share emotional concerns when the nurse did not engage. - Nurse-parent relationships was tempered by the company of the child. - Nurses responded to parents' emotions when openly expressed. More overt = the nurse was more likely to respond.
**Pecanac 2019**	Nurse-family communication during and after family meetings in the intensive care unit	United States	Explore nurse-family communication during and after family meetings	Qualitative - Conversation analysis	None	Recording of family meeting	n=36 family meetings	- Nurses only present in 15/36 meetings and spoke during 10 of the meetings. - Nurses rarely asked by family or healthcare professionals to contribute to the conversation. Nurses self-selected as the speaker by: (a) interrupting the current speaker, (b) collaboratively completing the current speaker’s turn, (c) responding to questions raised generally, and (d) asking a question during a pause.
**Trotta 2020**	Moving beyond the measure: Understanding patients' experiences of communication with nurses	United States	Better understand patients' perception of their communication with nurses	Qualitative - Content analysis	None	Interviews	n=49 patient interviews	- Misalignment between HCAHPS questions and participants' experiences (Cross-cutting responses, Verbal communication, Nonverbal communication) - Behaviors associated with effective nurse communication (Engagement, Anticipation, Responsiveness, Teaching) - Salient episodes during which nurses' behaviors resonated most (Nighttime, Painful and/or invasive procedures, Embarrassing moments)
**Adams 2014**	Nursing strategies to support family members of ICU patients at high risk of dying	United States	Explore how family members of intensive care patients at high risk of dying respond to nursing communication	Qualitative - Content analysis	None	Participant observation Interviews Document review	17 cases 42 interviews with 32 family members	Family members described five nursing approaches (Demonstrating concern, Building rapport, demonstrating professionalism, providing factual information, supporting decision-making)
**Alshammari 2022**	Adult patient communication experiences with nurses in cancer care settings: a qualitative study	Saudi Arabia	Explore the patient communication experiences with nurses in oncology care settings	Qualitative - Thematic analysis	Patient-centered communication in cancer care model (R. M. Epstein et al., 2005)	Interviews	n=21 patients with cancer NOT receiving palliative care 7 family members participated with the patiets	- The importance of patient-nurse relationships (nurses responsiveness, kindness, respectfulness, and politeness, trustworthiness) - Providing appropriate information to patients (exchanging information, decision-making) - Responding to patients emotional needs (doubts/fears/uncertainties, non-verbal support, psychological support) - Verbal communication between nurses and cancer patients (language barriers, communication strategies)
**Clayton 2014**	Communication behaviors and patient and caregiver emotional concerns: a description of home hospice communication	United States	Identify and describe communication behaviors used by hospice nurses when eliciting and addressing concerns with patients and caregivers	Qualitative - Thematic analysis	Patient-centered communication	Analysis of recorded nurse-patient/caregiver interactions	n=14 (7 patient/ caregiver dyads) 5 nurses	- Nurse communication behaviors reflecting positive emotional statements (e.g., compliments, optimism) - Frequent use of emotionally responsive statements to develop rapport with family – indicate hospice nurses use these communication behaviors to elicit issues responsible for emotional distress and indicate receptiveness to concerns.
**Forbes 2022**	From anticipation to confidence: A descriptive qualitative study of new graduate nurse communication with physicians	United States	Understand how new graduate nurses experience communication with physicians	Qualitative - Descriptive	None	Interviews	n= 14 nurses that had less than 2 years experience and worked in setting where communication with resident physicians occurred daily.	- Themes: Gaps in preparation, developing confidence, learning to communicate, interprofessional patient care. - Nurses develop skills at collaborating with physicians using reality-based situations - New graduate nurse knowledge of communicating with physicians develops through storytelling with other students, colleagues, and nursing instructors, which is void of any actual experience, creates anxiety and fear.
**Forbes 2020**	Getting work done: a grounded theory study of resident physician value of nursing communication	United States	Explore physician valuing of nursing communication in the context of patient care	Qualitative - Constructivist grounded theory	Relational Coordination Theory (RCT)	Interviews	n=15 internal medicine resident physicians	Theory of Getting Work Done derived from descriptions of how the resident physician *uses* the nurse to get the physician's work done. 1. Discerning the team: – Working separately – Developing trust 2. Shifting communication: – Supportive communication – Divisive communication – Directive communication 3. Accessing nurse knowledge and abilities – Constant presence – Compelling orders
**Holm 2021**	Nurses' experiences of serving as a communication guide and supporting the implementation of a communication intervention in the intensive care unit	Denmark	Explore the experience of serving as a nurse communication guide – supporting bottom-up implementation of multi-component communication intervention in the intensive care unit	Qualitative	Ricoeur hermeneutic phenomenology Medical Research Council's framework for developing complex interventions in healthcare	Interviews	n=8 nurses who volunteered to serve as communication guides. 15 interviews	- The communication intervention components provided overview, a conceptual framework, awareness and room for reflection - Being a communication guide illuminated the barriers and challenges of implementation - Communication interventions should be a guides while remaining adaptable to the needs of each specific situation
**Hudson 2019**	Addressing cancer patient and caregiver role transitions during home hospice nursing care	United States	Describe patient-caregiver-nurse communication during transitions at end of life	Qualitative - Content analysis	None	Secondary analysis of transcripts	n=19 home hospice visits 7 hospice agencies	- Negotiating transitions in patient independence - Navigating caregiver/patient emotions (e.g. frustration, sadness) through responses to transition conflict - Responses to emotional conflict included validation and reassurance.
**Kelly 2023**	Knowledge mobilization in critical care and the evolving communication role of nurses	United Kingdom	Understand the communication roles practiced by the critical care nurses pre-pandemic – appreciate preparedness and the impact these communication roles have upon colleagues, patients/family members	Qualitative - Thematic analysis	None	Interviews Focus group	n= 67, (46 critical care workers, 21 patients and family members)	- Team member: focused on staff collaboration and the impact this has on their working environment and the patient/family CC experience. - Diplomat: requires the nurse to provide conflict resolution for any of the disputes that arise between the doctors and the patient/family members. - Advocate: ensure that patients/family members understand medical decisions and communicate with colleagues the needs and wishes of the patient/family - Translator: interpreting complex medical information for their day-to-day practice and helping the patient/family in understanding their hospital experience - Friend: the role nurses play when moving away from discussing surrounding survival or the workings of the hospital environment and, instead discuss more mundane aspects of daily living.
**Li 2020**	General phenomenon and communication experience of physician and nurse in night shift communication: A qualitative study	China	Explore the phenomenon and psychological experience of communication in night shift medical staff – provide better reference for night shift communication between doctors and nurses	Qualitative	Husserl's phenomenology	Interviews	n= 13 8 nurses 5 doctors	- Theme: The need to achieve goals in night-time physician-nurse communication: - Theme: Obstacles in night-time physician-nurse communication and the responsibility is still very heavy - Theme: Relationship culture in night-time physician-nurse communication
**Montgomery 2017**	Communication During Palliative Care and End of Life: Perceptions of Experienced Pediatric Oncology Nurses	United States	Describe nurses perceptions of communicating during palliative care discussions, including perceptions of barriers and facilitators to effective communication.	Qualitative	Empirical phenomenology Group-as-a-whole theory	Focus group	12 focus groups 27 registered nurses with more than 5 years of experience or who were advanced practice nurses	- Evidence of continued evolution (reactive to proactive communication and care planning) - Skill of Knowing: Readiness to engage in palliative care discussions, supporting the child/family during palliative care discussions - Expanded Essence of Caring: Building intimate moments and fostering connectedness, balancing messages of hope and realism - Experienced Nurse as Committed Advocate: Parents are appreciative of knowing, Creative problem solver, Communication before a crisis - Valuing Individual Response to Grief: Culture of grief is experienced differently, experienced nurses need support too, supporting novice nurses
**Oliver 2019**	Behind the doors of home hospice patients: A secondary qualitative analysis of hospice nurse communication with patients and families	United States	Examine hospice nurses use of validation communication techniques	Qualitative	Dialectical Behavior Therapy	Audio recordings of hospice care visits	n=65 hospice nurses. No two visits were conducted by the same nurse	- Level 1: Paying attention - making an effort to hear and understand what is being communicated = 73% of validating comments. - Level 2: Reflecting back - restate the feelings that have been communicated. 19% of validating comments. - Level 3: Reading minds - identify something the patient or caregiver has not explicitly communicated. 4.5% of validating comments. - Level 4: Understanding in context - A message is communicated that the other person's reaction has a cause or makes sense given that person's diagnosis, history, or other relevant factors. 0.8% of validating comments. - Level 5: Recognizing the valid - A message is communicated, acknowledging that a person's thoughts, feelings, or behaviors are normal and understandable. 1.4% of validating comments. - Level 6: Showing equality - A message is communicated that the patient or caregiver is an equal. 0.8% of validating comments.
**Paulsen 2023**	Gynecological cancer survivors' experiences with sexual health communication in nurse-led follow-up consultations	Norway	Gain knowledge of gynecological cancer survivors experience of sexual health communication in nurse-led follow-up consultations	Qualitative	Gadamer's hermeneutic philosophy	Interviews	n=17 patients	- Importance of nurse listening to women's perception of sexuality - Post-treatment sexual challenges influence women's need to communicate about sexual health - Nursing communication can help women regain sexual health
**Simonovich 2021**	Examining effective communication in nursing practice during COVID-19: A large-scale qualitative study	United States	Examine qualitative communication experiences of nurses during the first wave of the COVID-19 pandemic in the United States	Qualitative - Thematic analysis	None	Interviews	n=100 nurses	Importance of effective communication including presence, education and emotional support across three levels: Organizational leadership, unit leadership, nurse-to-nurse leadership
**Wittenberg 2017**	Exploring Nurse Communication About Spirituality	United States	Explore the spiritual care experiences of oncology nurses in order to learn more about patient needs and nurse responses.	Qualitative - Thematic analysis	None	Review of written summary of a spiritual care experience with a patient or family member.	n=57 nurses 36.8% clinical nurses 19.3% advanced practice nurses 25% nurse managers, educators, or administrator	- Initiating communication about spirituality: patients rather than family members usually initiate such communication, and it generally occurred around patients impending deaths - Responding to topics about spirituality: nurses frequently performed spiritual work by responding to patients‚ family members‚ religious/spiritual crises. - Sharing personal spiritual background and religious preferences: Nurses own spiritual lives frequently were invoked as they attended to their patients spiritual stories, questions, and crises.
**De Rezende 2015**	Body language in health care: a contribution to nursing communication	Brazil	To classify body language used in nursing care and propose “body language in nursing care” as an analytical category for nursing communication	Quantitative – systematic observation	None	Systematic observation of 21:43 care situations	n=21 nurses 43 care situations 2 hospitals	- Sound expressions emphasized laughter. - Facial expressions communicated satisfaction and happiness - Eye contact with members stood out in visual expressions. - The most frequent body expressions were head movements and indistinct touches - Nursing care team members use body language to establish rapport with patients, clarify their needs and plan care.
**Kim 2020**	Communication skills, problem-solving ability, understanding of patients’ conditions, and nurse’s perception of professionalism among clinical nurses: a structural equation model analysis	South Korea	Confirm the structural relationship between clinical nurse communication skills, problem-solving ability, understanding of patients’ conditions, and nurse’s perception of professionalism.	Quantitative – Structural equation analysis	None	Frequency analysis, identification factor analysis, reliability analysis, measurement model analysis, model fit, intervention effects.	n=171 nurses 3 cities	- Nurse’s perception of professionalism was influenced by factors of communication skills and understanding of the patient’s condition – not by their ability to solve problems. - Understanding of patient’s condition had a mediating effect on communication skills and nursing awareness. - Communication skills and understanding of the patient’s condition greatly influenced the nurse’s perception of professionalism.
**Myers 2020**	Nurses’ active empathetic listening behaviors from the voice of the patient	United States	Identify how adult hospitalized patients perceive effective and ineffective nurse active empathetic listening behaviors	Quantitative – two group comparative descriptive study	None	Independent t-test (two-tailed) for unequal variances was used for all but two of the AEL scale questions to determine if there was a significant difference between the two groups	n = adults who experienced inpatient acute care hospitalization and were discharged from a preselected med-surg unit.	- No significant differences were noted in the demographics between those participants who perceived their nurses listened to them throughout their hospitalization - This study suggests effective active empathetic nurse listening skills will influence a positive patient experience
**Reblin 2019**	Communication of emotion in home hospice cancer care: implications for spouse caregiver depression into bereavement	United States	Identify the effects of hospice nurse supportive communication and caregiver-nurse exchange of positive emotions on family caregiver depression during bereavement	Quantitative	None	Digitally recorded nurse home visit conversation. Caregiver completion of Hospital anxiety and depression scale anxiety subscale and geriatric depression scale-short form.	n=58 hospice nurses N=101 family caregivers of cancer patients 10 hospice agencies	- Multilevel modeling revealed that caregiver positive emotion communication and nurse emotional response communication are associated with caregiver depression in bereavement - No significant association between caregiver distress communication and depression in bereavement
**Saraswasta 2021**	Implementation of effective nurse communication in hospital through electronic nursing documentation	Indonesia	Identify the implementation of effective communication of nurses in hospitals through electronic nursing documentation	Qualitative – cross sectional design	None	Effective communication instrument (52 statements)	n=243 nurses	- Implementation of effective communication of nurses was 80.18% of the maximum value - Highest effective communication occurred on patient discharge while the lowest conducted in receiving messages by phone - Implementation of effective communication during the nursing process is 78.70%
**Wittenberg 2018**	Health literacy: exploring nursing challenges to providing support and understanding	United States	Explore nurse communication and patient health literacy	Quantitative – Cross sectional design	COMFORT framework	Open-ended survey distributed to nurses attending a COMFORT communication training course	n=74 oncology nurses	- Majority of the nurses reported communication challenges with patients who spoke English as a second language - Nurses were least comfortable identifying low-literacy patients and assessing a patient’s health literacy level - More experienced nurses reported more difficulty with low-literacy populations than less experienced nurses
**Wune 2020**	Nurses to patients communication and barriers perceived by nurses at Tikur Anbessa specialized hospital	Ethiopia	Assess level of nurse-patient communication and its barriers among nurses in the study area	Quantitative – Cross sectional design	none	Institutional cross sectional design Semi structured questionnaire	n=296 nurses	- The level of effective nurse-patient communication in this study = 34.5%. Lack of time and work overload were predominantly reported by nurses as the main barriers to effective nurses-patient communication - Time adequacy, engaging in multiple jobs and fatigue and family and friends at bedside were factors associated with patient-nurse communication
**Höglander 2023**	Registered nurse-patient communication research: an integrative review for future directions in nursing research	Sweden	Explore communication research in nursing by investigating the theoretical approaches, methods, content and perspectives in research on real-time registered nurse (RN)–patient communication	Integrative review	none	integrative review of real-time communication between RNs and patients	n=52 articles	- The integration of theories is weak in most included studies. - nurse–patient communication appears to influence relationship building. - Even when nurses strive to meet patients' needs, they often focus primarily on nursing routines and physical care. - The topic of the communication varies depending on the situation and different communication styles are used. - How the nurses communicated subsequently influenced the patients' communication styles and strategies - Patients were often vague or unclear in expressions of their concerns, making communication skills of the nurse more important - Including family & loved ones promoted trust in the nurse-patient relationship - Communication regarding physical care far outweighed psychosocial issues, positive emotions, and partner statements - Nurse communication is often embedded in everyday activities
**Kerr 2020**	The effectiveness of training interventions on nurses’ communication skills: a systematic review	Australia	Identify, critically appraise and synthesize evidence for the effectiveness of communication skills training interventions in nursing practice	Systematic review	None	PRISMA	n=7 randomized control trials	- Direct impact of educational interventions on nurses’ communication skills difficult to measure arising from non-standardized outcome measurement tools - Majority of research focused on cancer patients - All communication training reporter at least 1 positive effect - Subjective self-assessment of communication skills prone to bias and overestimate abilities – need to be paired with robust objective measurement tools - Nurses more likely to use patient-centered communications techniques after communication training - Organization-wide communication training is the most effective but the most difficult to implement, especially in low resource organizations and countries - When planning evaluative studies, measurement tools should be selected or developed based on target health outcomes
**Gutiérrez-Puertas 2020**	Educational interventions for nursing students to develop communication skills with patients: a systematic review	Spain	Know the impact of educational interventions on nursing students to develop their communication skills with patients	Systematic review	Therapeutic communication	Systematic review PRISMA	n=19 studies	- Only 2 studies used theoretical frameworks to guide interventions - Numerous definitions exist to refer to patient-centered communication (therapeutic communication, nurse-patient communication, or interpersonal communication) - Validity and reliability lacking for patient-centered communication measures - Debriefing and the opportunity to get and provide feedback after a communication simulation allowed nursing students to improve later performance - Context mainly based on mental health, end-of-life, and maternity. - Simulation was the most common method

### Synthesizing argument

**Fig. 3:**
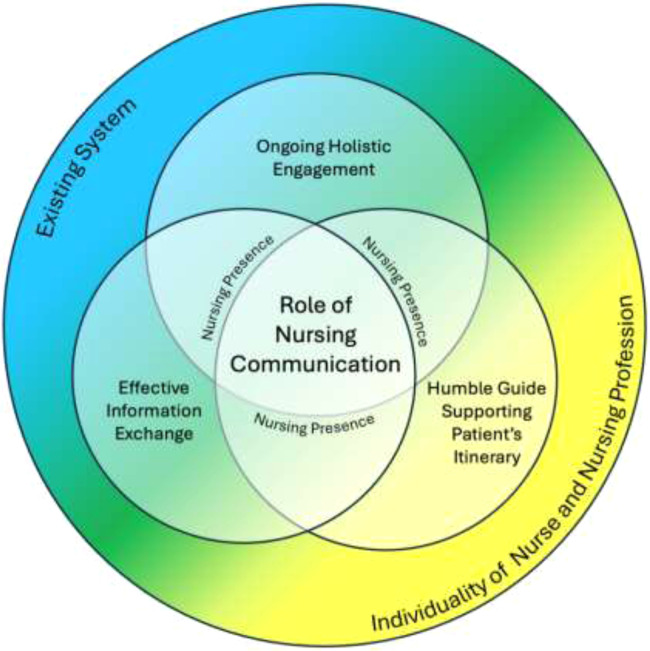
Conceptual framework for the role of nursing communication in patient care.

*Note.* The large primary circle represents the individuality of the nurse and the nursing profession (yellow) interacting with existing health organizations or systems (blue). The role of nursing communication develops at this juncture (green). Within the larger circle are the intersecting constructs of ongoing holistic engagement, effective information exchange, and humble guide supporting the patient's itinerary. Nursing presence is in multiple overlapping circles to represent its broad impact on all other constructs. *The role of nursing communication forms from and within these constructs*.

Based on the literature synthesis, five synthetic constructs emerged that clarified the role of nursing communication in patient care and meaningfully contributed to the final conceptual framework: 1) ongoing holistic engagement, 2) humble guide supporting the patient's itinerary, 3) effective information exchange, 4) maintaining nursing individuality within an existing system, and 5) nursing presence. These synthetic constructs guided the following synthesizing argument: *the role of nursing communication in patient care emphasizes maintaining a nursing presence that communicates high-quality physical care and ongoing holistic engagement centered around the patient's values, needs, and wishes* (Fig. 3).

### Conceptual framework

The following conceptual framework (Fig. 3) represents the relational links between synthetic constructs, explains their associations, and provides formalized and generalizable ways of understanding the role of nursing communication (Dixon-Woods et al., 2006; Flemming, 2010).

### Ongoing holistic engagement

Nurses consciously and unconsciously communicate with their patients through ongoing holistic engagement that, ideally, provides comfort, reassurance, and hope in complex and variable patient environments. Communication during physical care allows the patient to feel appreciated as a whole person, beyond solely their physical body. Ongoing holistic engagement facilitates a nurse-patient connection that *engages patients in conversations* (Adams et al., 2014; Hudson et al., 2019; Kelly et al., 2023; Oliver et al., 2019; Pecanac and King, 2019; Trotta et al., 2020; Wittenberg et al., 2017)*, anticipates patient needs* (Adams et al., 2014; Alshammari et al., 2022; Clayton et al., 2014; Crawford et al., 2017; Holm et al., 2021; Trotta et al., 2020)*, and reframes hope* (Adams et al., 2014; Crawford et al., 2017; Montgomery et al., 2017; Simonovich et al., 2021; Trotta et al., 2020).

**Engaging in Conversation.** Both small talk and deep, emotionally laden conversations have a place in providing holistic care. Yet, overwhelmingly, patients and families appreciate talking with their nurses about something other than their health. Bedside nurses often "discussed more mundane aspects of daily living [and] the role of friend was a lifeline for both patients and family members" (Kelly et al., 2023, p. 918). Holistic communication includes recognizing a patient as an individual outside the stereotypical patient roles. For the patient, their nurse’s engagement in humor, small talk, and self-disclosure recognized them as a whole person and more than just their disease process. While most nurses expressed an open willingness to engage in deep emotional conversations with their patients, few nurses or patients reported sentinel emotional moments. Rather, communication surrounding the joys and hardships inherent to a person's health was ongoing. The trust built was not indicative of a huge self-disclosure but a trust that the nurse would take care of them, trust built through the nurse's care, comfort, and responsiveness to their physical needs with warmth and kindness (Montgomery et al., 2017).

**Anticipation of Needs.** Nurses communicate with patients by proactively attending to physical needs and fluctuating emotions. By getting to know their patients, nurses recognize subtle patient concerns or distress and proactively respond with the appropriate verbal and nonverbal communication (Trotta et al., 2020). "When I needed that extra pat on the shoulder or that extra look across the room, he/she seemed to be very in tune with that and that was just that connection" (Adams et al., 2014, p. 409). Anticipating needs also includes anticipating change and the open recognition that a patient's life, health, and illness are complex and variable (Holm et al., 2021). Furthermore, patients desire this connection and hope to maintain it through continuity in their nursing care (Crawford et al., 2017).

**Reframing Hope.** Nurses often discussed helping patients and family members reframe their concept of hope when faced with a new diagnosis or prognosis, and "although family members were hungry for information, they also highly valued…the nurses ability to deliver information in a way that supported hope" (Adams et al., 2014, p. 412). Nurses are attuned to when patients or family members may be emotionally ready to discuss reframing their hope, "you can't predict when they're going to be ready for that conversation. It just sort of happens. I mean opportunity knocks" (Montgomery et al., 2017, p. E50). Part of reframing hope was explicitly addressing feelings of uncertainty (Crawford et al., 2017; Trotta et al., 2020). Addressing uncertainty came in many forms, from providing clarifying information to simply acknowledging how difficult the uncertainty of a disease was and validating those feelings.

### Humble guide supporting the patient's itinerary

Nurses provide communication that guides patients through their health and wellness journeys. The phrase *humble guide* represents how the role of nursing communication needs to account for individual and cultural variations, and *supporting the Patient's Itinerary* represents how nurses communicate with an understanding that patients are the subject matter experts on their feelings, beliefs, and values. Therefore, any guidance provided must first humbly acknowledge the patient's expertise. Being a humble guide supporting the patient's itinerary builds on the premise that patients are the ones who set their health and wellness goals. Nurses who take on this role communicate in a way that *fosters an environment of cultural safety* (Adams et al., 2014; Alshammari et al., 2022; Holm et al., 2021; Kelly et al., 2023; Montgomery et al., 2017; Trotta et al., 2020) for the patient and unobtrusively *mediate conversations toward their patient's wishes* (Clayton et al., 2014; Crawford et al., 2017; Holm et al., 2021; Hudson et al., 2019; Montgomery et al., 2017; Pecanac and King, 2019).

**Foster Cultural Safety.** A central role of nursing communication is fostering cultural safety. Nurses have disproportionately higher communication opportunities with patients compared to other healthcare providers, making this role all the more critical.

Cultural safety encompasses a critical consciousness where healthcare professionals and healthcare organizations engage in ongoing self-reflection and self-awareness and hold themselves accountable for providing culturally safe care, as defined by the patient and their communities, and as measured through progress towards achieving health equity. (Curtis et al., 2019, p. 14).

Nurses openly recognize the importance of fostering cultural safety for their patients while simultaneously expressing how challenging it is to communicate with patients and families from different cultures. Nurses discussed being receptive to the "patient/family as in-the-moment, focused, and non-judgmental. Even if they did not share the patient/family beliefs, they encouraged the expression of those beliefs" (Wittenberg et al., 2017, p. 568). While not easy, nurses foster cultural safety by acknowledging their own potential bias and working towards remaining unbiased in decision-making (Adams et al., 2014; Montgomery et al., 2017).

**Mediate Toward Patient’s Wishes.** Often, many well-intentioned but competing desires, cultures, and ideas intersect in the course of patient care. A familiar role of nursing communication is mediating these differences while always bringing the patient's wishes and perspective to the forefront of any discussion. Crawford et al. (2017) used the term *cultural broker* to describe how nurses are placed in intermediary roles that negotiate and intervene between the hospital and patient cultures. The parties involved in these mediations are as variable as the patients. Sometimes, the nurse will facilitate discussions between the patient and their loved ones, and at times, the nurse will prompt the patient to voice their concerns to physicians. During these discussions, nurses recognize the significance of balance and flexibility (Holms et al., 2014). While nurses rarely spoke of setting up formal mediations, there were specific situations where nurses provided more direct guidance, such as reassuring loved ones through a patient's imminent death (Montgomery et al., 2017).

### Effective information exchange

Effectively exchanging information, knowledge, and logistical updates is vital for accurate and timely medical care and, therefore, an essential role of nursing communication. Much of the verbal communication people associate with nursing communication falls into this construct. Effective information exchange includes hand-off reports, notifying physicians of changes in patient status, and formally educating patients on new medications or disease processes. Achieving this information exchange involves effectively exchanging *factual and logistical information* (Adams et al., 2014; Alshammari et al., 2022; Forbes and Evans, 2022; Forbes et al., 2020; Holm et al., 2021; Hudson et al., 2019; Kelly et al., 2023; Li et al., 2020; Montgomery et al., 2017; Pecanac and King, 2019) and *translating* that information based on the unique individual and context (Adams et al., 2014; Crawford et al., 2017; Forbes and Evans, 2022; Forbes et al., 2020; Kelly et al., 2023).

**Factual and Logistical Information Exchange.** The role of nursing communication includes exchanging knowledge effectively in a way that all people involved understand and comprehend. Interdisciplinary team members rely on nurses for informative updates on a patient's health status and clear documentation of their nursing care (Forbes and Evans, 2022; Forbes et al., 2020; Holm et al., 2021). Patients and families also ask nurses about everything from who is going to walk their dog while they are in the hospital to legal issues such as changing documentation of ownership (Hudson et al., 2019). While the role of nursing communication cannot and should not imply that nurses have the answers to all these logistical questions, there is an expectation that nurses know how to address them by connecting the patient or family with the appropriate interdisciplinary team member, such as a case manager, social worker, spiritual advisor, or physician.

**Translator.** When patients do not understand their health status, disease, or treatment plan, their uncertainty increases along with feelings of anxiety and discomfort. Therefore, an important role of nursing communication is *translator.* Nurses act as translators by ensuring that patients and family members understand information from other healthcare providers and bring medical jargon to their patient's level of understanding (Kelly et al., 2023). Nurses in the intensive care unit, recognizing their role as translators, often stay behind with the patient and family after an interdisciplinary team meeting to ensure they understand what was said (Forbes and Evans, 2022). Nurses also translate information based on the needs of interdisciplinary team members. For example, nurses report translating information regarding their patient's needs, preferences and wishes into concise statements with clear *asks* for the on-call physician. Then, rephrasing the physician's response into language accessible to a non-health professional when returning to the patient (Alshammari et al., 2022; Forbes and Evans, 2022; Kelly et al., 2023).

### Maintaining nursing individuality within an existing system

A role of nursing communication is integrating into an existing organization in a way that *maintains and empowers the individuality of oneself and the nursing profession* (Adams et al., 2014; Forbes and Evans, 2022; Forbes et al., 2020; Holm et al., 2021; Kelly et al., 2023; Li et al., 2020; Montgomery et al., 2017; Pecanac and King, 2019; Simonovich et al., 2021) while practicing within *existing workplace cultures and interdisciplinary hierarchies* (Alshammari et al., 2022; Crawford et al., 2017; Forbes and Evans, 2022; Forbes et al., 2020; Kelly et al., 2023; Li et al., 2020; Montgomery et al., 2017; Pecanac and King, 2019; Simonovich et al., 2021; Trotta et al., 2020).

**Existing Workplace Culture and Interdisciplinary Hierarchies.** Workplace culture and institutional hierarchies highly influence the communicatory role nurses enact. Furthermore, limited time and resources in hospital work environments impact the entire healthcare team and their subsequent communication strategies (Forbes et al., 2020). Studies outside the US and Western Europe demonstrated an expectation that physicians are the only people who should present medical information to patients, and emotional support is primarily sought from one's family (Alshammari et al., 2022; Li et al., 2020). However, the nurse's presence is still recognized by patients in those hospital systems, where patients voiced feeling comforted and reassured by the nurse regularly checking in on them, providing high-quality physical care, and exuding a warm and kind demeanor (Alshammari et al., 2022; Li et al., 2020).

Workplace culture and hierarchies include how other interdisciplinary team members perceive nursing. When physicians address nursing communication, they often do so from the perspectives of their own needs and associate nurse competence solely with their ability to complete physician orders (Forbes and Evans, 2022; Li et al., 2020). Since nurses want to be perceived by the physicians as competent, they may base their communication around the physician's comfort before the patient’s or their own. Conversely, healthcare systems that foster equality in interdisciplinary relationships empower nursing communication and autonomy (Simonovich et al., 2021).

**Empower Nursing's Unique Communicatory Role.** The role of nursing communication incorporates and embraces the uniqueness of the nurse instead of attempting to standardize the nurse to fit specific communication interventions. Nurses are as individual as the patients they care for and bring unique life experience, work experience, educational backgrounds, personalities, limitations, and expertise to their role (Holm et al., 2021). Therefore, nurses often rely on their prior experiences to evaluate how to navigate and tailor patient communication (Kelly et al., 2023). Remaining open to learn and grow is vital at every stage of a nurse’s career. However, growth does not replace an individual’s positive or negative experiences but builds upon them (Holm et al., 2021). Therefore, nurses' valuable experience and expertise must always be considered when developing future communication frameworks and theories.

Several nurses expressed the need for self-communication regarding their own wellbeing and turning to other nurses for support (Montgomery et al., 2017; Simonovich et al., 2021). There will always be times when nurses feel at a loss for how to best communicate, regardless of education or experience. "This loss was often accompanied by the nurse's own fears and insecurities about saying the right thing" (Montgomery et al., 2017, p. E53). During these times, nurses articulate the importance of communicating one's own feelings, concerns, and needs.

### Nursing presence

Nurses communicate their concern and support by being physically and metaphorically present for their patients (Adams et al., 2014; Alshammari et al., 2022; Clayton et al., 2014; Crawford et al., 2017; Holm et al., 2021; Hudson et al., 2019; Kelly et al., 2023; Montgomery et al., 2017; Paulsen et al., 2023; Pecanac and King, 2019; Simonovich et al., 2021; Trotta et al., 2020; Wittenberg et al., 2016). Nursing presence formed as a synthesizing construct later in the synthesis process with the recognition that a *nursing presence* was either explicitly or implicitly identified throughout all preceding constructs. The nurse's ongoing presence creates many smaller, intimate communicatory moments that build upon each other to foster connectedness (Montgomery et al., 2017). This nursing presence provides hope and reassurance during the patient's sentinel moments, such as fearful or embarrassing episodes. "Even at night, in the middle of the night, sometimes I couldn't sleep, and I'd see them. They'd peek in to see how I was doing, so I really appreciated that" (Trotta et al., 2020, p. 575). Nurses and patients discussed the nurse *giving space while being present*. In these times the patient and family were comforted by knowing that the nurse would come if needed but also knew to give them space and time to be together while processing new information. Patients often felt their nurse’s presence in conjunction with perceiving that the nurse had listened intently to them (Trotta et al., 2020). Feeling heard and understood allowed the patient to continue to sense the nurse's presence even after they left the room (Paulsen et al., 2023).

## Discussion

The synthesizing argument formed from this Critical Interpretive Synthesis is that nursing communication emphasizes maintaining a nursing presence that communicates high-quality physical care and ongoing holistic engagement centered around the patient's values, needs, and wishes. This paper is about the role of nursing communication in patient care, not an assessment of how good or bad nurses are at that communication. Furthermore, this paper is not intended to diminish or discredit the valuable research findings surrounding effective patient-centered and relationship-centered communication techniques. Research shows these techniques often benefit nurses in communicating with patients, families, and interdisciplinary teams (Chou and Cooley, 2018; Kwame and Petrucka, 2021). However, with clarification surrounding the role of nursing communication, we can assess the effectiveness of these techniques in the context of nursing instead of having the technique define the nurse's role.

### Implication for nursing theory

None of the articles in this Critical Interpretive Synthesis were explicitly grounded in a nursing theory or philosophy, which is unsurprising because many health communication interventions, techniques, and frameworks were first developed for physician use. However, while "some communication skills described for physicians treating patients… may translate to nursing practice…the role nurses play is distinct enough that strategies specific to nurses are likely to be more effective" (Adams et al., 2014, p. 407). For example, the NURSE framework for responding to emotional cues was created by a physician from a physician's perspective (Ciarkowski, 2020). Instead of the term "nursing communication" the phrase "communication in nursing" is often used, exemplified by the two primary textbooks on the subject being titled *Communication in Nursing* (Riley, 2019) and *Communication for Nurses* (Sheldon and Foust, 2014). While this may seem like simple semantics with no meaningful difference between the two, the verbiage "communication in nursing" denotes communication research and theory conducted outside nursing being applied to nursing, while "nursing communication" encapsulates the communication nurses provide as unique to the discipline of nursing, requiring its own theory and research. Therefore, future research needs to focus on developing frameworks within nursing instead of solely applying outside frameworks.

### Implications for nursing education

The construct of *humble guide that supports a patient's itinerary* emphasizes supporting the patient's cultural safety. However, the concept of cultural safety highlights the need for interventions that extend beyond communication education and emphasize the importance of greater representation within the nursing workforce. Although none of the reviewed articles explicitly addressed culturally specific definitions of communication, several studies reported that patients felt more comfortable when cared for and communicated with by nurses who shared a similar cultural background. When nurses’ spiritual or cultural beliefs aligned with those of their patients, both parties reported a stronger sense of connection grounded in shared experiences and values(Alshammari et al., 2022; Wittenberg et al., 2017). Improving representation in nursing starts with our nursing schools. Therefore, while we need to develop educational syllabi that teach and promote the role of nursing communication in patient care, we also must improve access to nursing education for all cultures, races, gender identities, and socioeconomic statuses (Gordon and Patterson, 2023; Patterson et al., 2023). Social determinants of health impact our patients and nurses when they become barriers to accessing quality nursing education (Garcia et al., 2021).

### Implications for nursing practice

Much of the role of nursing communication centers around the nurse being present in the patient's life, which provides the opportunity to connect, care, and support. However, if every minute of a nurse's time and communication is "optimized" for "effective nursing communication" as defined by outcome-specific goals such as quality indicators, time management, and HCAHP scores, the role of nursing communication will be lost within healthcare systems driven by financial reimbursement. Nurses highlighted interdisciplinary communication as one of their most significant sources of tension and feelings of disrespect when their concerns and insights were ignored (Kelly et al., 2023; Montgomery et al., 2017). While providing unique insight to the interdisciplinary team is absolutely part of nursing communication, hierarchical interdisciplinary structures in our healthcare systems disempower nurses, preventing them from taking a more active role as patient advocates during conversations (Pecanac and King, 2019).

Implementing changes in communication behaviors is extremely difficult and likely to fail if the nurse is expected to change or ignore fundamental aspects of themselves. Communication is both learned and innate, combining a blend of learnable skills with the inherent qualities of the individual nurse (Montgomery et al., 2017). Adams (2014) critiques nurses' reliance on intuition or personal preferences to guide their communication. However, this intuition and preference is built upon experience and personality, neither of which can be removed (Holm et al., 2021). "Changing communication in a professional context may be difficult because the nurse then needs to change his or her personal trait or style of communication." (Holms et al., 2014, p. 8). Nurses recognize the importance of improving communication skills that promote patient care and are enthusiastic about learning those new skills. Yet, they still report struggling to change ingrained communication behaviors (Holm et al., 2021). Therefore, changing routines and communication culture is much more difficult and complex than providing education sessions on communication techniques (Holm et al., 2021).

### Limitations

Reproducibility is limited to the methodological design of this synthesis and not its findings because Critical Interpretive Synthesis results are highly dependent on the author's interpretation (Depraetere et al., 2021). The primary author of this study has worked as a bedside nurse and nurse manager for over eight years and is actively researching unique aspects of the nurse-patient relationships, which necessarily impact the interpretive findings. Assessing the availability and effectiveness of tools and strategies to enhance nursing communication was beyond the scope of this Critical Interpretive Synthesis but warrants further review and research. No review protocol was registered for this study's PRISMA literature review. Like any new conceptual contribution, this framework will benefit from further refinement and testing by the nursing research community.

## Conclusion

The role of nursing communication in patient care emphasizes maintaining a nursing presence that communicates high-quality physical care and ongoing holistic engagement centered around the patient's values, needs, and wishes. While the importance of nursing communication is widely recognized, outside disciplines and organizational systems have largely defined this role. With this conceptual framework we now have a distinctly nursing perspective on the role of nursing communication. This perspective prioritizes the unique nurse-patient communicatory relationship and gives nurse researchers, policymakers, and educators a new way of thinking about and prioritizing the role of nursing communication.

## Ethics approval and consent to participate

Ethics approval and consent to participate are not applicable because this synthesis is based exclusively on published literature.

## Availability of data and materials

The data analyzed during this Critical Interpretive Synthesis, including the reciprocal translation and integrative grid, are available from the corresponding author upon reasonable request.

## CRediT authorship contribution statement

**Julie B. Grant:** Writing – review & editing, Writing – original draft, Methodology, Formal analysis, Conceptualization. **Jacqueline Jones:** Writing – review & editing, Supervision, Conceptualization. **Carey Candrian:** Writing – review & editing, Supervision. **Kathleen S. Oman:** Writing – review & editing, Supervision. **Sean M. Reed:** Writing – review & editing, Supervision, Methodology, Conceptualization.

## Declaration of competing interest

The authors declare that they have no known competing financial interests or personal relationships that could have appeared to influence the work reported in this paper.
